# Metal Complexes of 1,3,4-Thiadiazole-2,5-Disulfonamide are Strong Dual Carbonic Anhydrase Inhibitors, although the Ligand Possesses very Weak such Properties

**DOI:** 10.1155/MBD.1995.331

**Published:** 1995

**Authors:** Claudiu T. Supuran

**Affiliations:** 1 University of Florence Laboratory of Inorganic and Bioinorganic Chemistry Via Gino Capponi 7 Firenze 1-50121 Italy

## Abstract

Coordination compounds of Co(II), Ni(II), Cu(II), Zn(II), and Cd(II) with 1,3,4-thiadiazole-2,5-disulfonamide as ligand were synthesized and characterized by IR and UV spectroscopy, conductimetry and thermogravimetry. The parent ligand is a very weak carbonic anhydrase (CA) inhibitor, although it constituted the lead for developing important classes of diuretics. The complex derivatives behave as much stronger CA inhibitors, with IC_50_ values around 10^−8^M against isozyme CA II, and 10^−7^ M against isozyme CAI.


### METAL COMPLEXES OF 1,3,4-THIADIAZOLE-2,5-DISULFONAMIDE ARE STRONG DUAL CARBONIC ANHYDRASE INHIBITORS, ALTHOUGH THE LIGAND POSSESSES VERY WEAK SUCH PROPERTIES

Claudiu T. Supuran
University of Florence, Laboratory of Inorganic and Bioinorganic Chemistry,
Via Gino Capponi 7, I-50121, Firenze, Italy

**Abstract**: Coordination compounds of Co(II), Ni(II), Cu(II), Zn(II), and Cd(II) with 1,3,4-thiadiazole-2,5-disulfonamide as ligand were synthesized and characterized by IR and UV spectroscopy, conductimetry and thermogravimetry. The parent ligand is a very weak carbonic anhydrase (CA) inhibitor, although it constituted the lead for developing important classes of diuretics. The complex derivatives behave as much stronger CA inhibitors, with $IC_{50}$ values around $10^{-8}$ M against isozyme CA II, and $10^{-7}$ M against isozyme CA I.

### Introduction

Sulfonamide inhibitors of the zinc enzyme carbonic anhydrase (CA, EC 4.2.1.1) are widely used pharmacological agents in the treatment of a variety of disorders. [1-4] They include derivatives such as acetazolamide **1**, methazolamide **2**, as well as the recently developed thienothiopyran sulfonamides **3** introduced in clinical medicine as successful topical antiglaucoma drugs. [2,3]

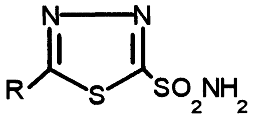

1: R= $CH_3CONH$

4: R= $SO_2NH_2$

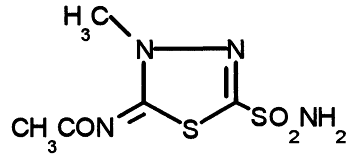

**2**

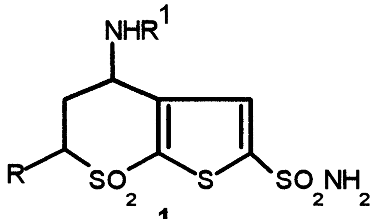

a: R=H; $R^1$= $Me_2CHCH_2$

3 b: R=Me; $R^1$ = Et

It was recently reported by us [4-6] that the metal complexes of sulfonamides **1-3** behave as very strong CA inhibitors, and their mechanism of action has also been explained [7] as being due to a dual inhibition, by means of sulfonamide anions and metal ions, formed by dissociation of the complexes in dilute solutions during the enzymatic assay.[7] The sulfonamide anions bind thereafter to the catalytically vital Zn(II) ion within the CA active site, whereas cations probably bind in the neighborhood of active site residue His-64 (which acts as a proton shuttle during the catalytic turnover [8]), disturbing in this way the whole catalytic cycle.[4-7]

1,3,4-Thiadiazole-2,5-disulfonamide **4**, a compound reported in the classical study of CA inhibitors of Roblin and Clapp, [9] was considered to be a very potent inhibitor, and it constituted the lead for developing important classes of pharmacological agents such as the thiazide saluretics and the high ceiling diuretics. [3,10] Recently we reinvestigated [11] this compound and showed that it possesses unexpectedly weak CA II inhibitory properties, but we were unable to explain why the previous researchers obtained erroneous data.[9] What is more important is the fact that although a weak inhibitor, **4** was a good lead molecule, since it constituted the starting point for obtaining such widely used drugs as chlorothiazide or furosemide. [3,11]

As we are also interested in exploring the coordination chemistry of sulfonamide CA inhibitors, in addition to their pharmacological applications, [1,4,5,11] a study is reported here on the preparation and characterization of metal complexes containing 4 and divalent metal ions such as Co(II), Ni(II), Cu(II), Zn(II), and Cd(II). In fact 4 is the first disulfonamide for which such a study was done. Here we report the data on these new derivatives, as well as their inhibition properties against two isozymes, CA I and CA II. Mention should be made that CA I inhibition data for the parent ligand, 1,3,4-thiadiazole-2,5-disulfonamide are published here for the first time.

### Materials and Methods

Melting points were recorded on a heating plate microscope and are not corrected. FTIR spectra were obtained on thin films of pure compound, with a Perkin Elmer 1600 instrument, in the range 200 - 4000 $cm^{-1}$. Electronic spectra were obtained by the diffuse reflectance technique in MgO as reference, with a Perkin Elmer Lambda 17 apparatus. Conductimetric measurements were done in DMF solutions, at 25°C (concentrations of 1 mM of complex) with a Fisher conductimeter. $^1H$- and $^{13}C$-NMR data were obtained in DMSO-$d_6$ as solvent with a Varian Gemmini 300 spectrometer. Chemical shifts are expressed as δ values relative to tetramethylsilane as internal standard. Elemental analyses were done by combustion for C,H,N with an automated Carlo Erba analyzer, and gravimetrically for the metal ions, and were ± 0.4% of the theoretical values. Thermogravimetric measurements were done in air, at a heating rate of 10°C/min., with a Perkin Elmer 3600 thermobalance.

Sulfonamide 4 used in the syntheses was prepared as described earlier by us.[11] Metal salts, organic reagents used for preparing the ligand 4 and solvents were from Aldrich and were used without additional purification. Bovine CA II and human CA I was from Sigma Chemical Co. Inhibitors were assayed by Maren's micromethod [12], in the conditions of the E-I (enzyme-inhibitor) technique, at 0°C in veronal buffer. $IC_{50}$ values represent the molarity of inhibitor producing a 50% decrease of CA specific activity for the $CO_2$ hydration reaction.

### Synthesis of coordination compounds 5-9

An amount of 10 mMoles of sulfonamide 4 was converted into the disodium salt, by dissolving in a solution obtained from 20 mMoles of NaOH and 15 mL water. The disodium salt obtained in this way was treated then with a solution of metal salt ($MCl_2 .xH_2O$, where M = Co(II), Ni(II), Zn(II), Cd(II) and Cu(II) ), in ethanol, working at molar ratios sulfonamide : M(II) of 1:1. The complexes **5-9** precipitated immediately, were filtered and air dried. Yields were in the range of 50-60%.

### Synthesis of coordination compounds 10-14

10 mMoles sulfonamide 4 were suspended in 25 mL of concentrated ammonia solution and stirred energetically till complete dissolution, then 10 mMoles of $MCl_2$ (as above) dissolved in a small amount of water were added dropwise. The precipitated complexes were filtered and air dried. Yields were higher than for the previous complexes (70-75%).

### Results and Discussion

Two approaches were used for the preparation of complexes containing 1,3,4-thiadiazole-2,5-disulfonamide as ligand, both of which were widely employed before by us [6a,b,13] and by Borras' [6c,14] group for preparing acetazolamide 1 and methazolamide 2 complexes, some of which were subsequently characterized extensively by spectroscopic and X-ray crystallographic methods. [5] According to our method [6a] the coordination compounds of sulfonamides were prepared starting from the sodium salt of the ligand, in aqueous milieu, whereas according to Borras' group they were obtained in the presence of ammonia (or other amines, such as pyridine, ethylenediamine, imidazole, etc), in alcohol as solvent.[14] In such cases, the amines are also coordinated to the metal ions, in addition to the sulfonamide ligands. In the present study, we prepared and characterized both types of derivatives, working with the sodium salt of 4, but also with the ligand in the presence of ammonia, in both cases in a 1:1 molar ratio ligand:metal salt.

The new derivatives prepared in this study, containing 4 and the following metal ions, Co(II), Ni(II),Cu(II), Zn(II), and Cd(II), of type **5-14**, are shown in Table I, together with their elemental analysis data (within ± 0.4% of the theoretical values).

Table I: Complexes **5-14** prepared and their elemental analysis data (S stands for the doubly deprotonated species of sulfonamide **4**).

| No. | Compound | Color | Analysis (calc./found) %M[a] | %C[b] | %H[b] | %N[b] |
|---|---|---|---|---|---|---|
| **5** | $[CoS(OH_2)_2]_n$ | black-violet | 17.5/17..2 | 7.1/6.9 | 1.8/1.6 | 16.6/16.4 |
| **6** | $[NiS(OH_2)_2]_n$ | deep green | 17.4/17.1 | 7.1/7.1 | 1.7/1.5 | 16.6/16.5 |
| **7** | $[CuS(OH_2)_2]_n$ | olive | 18.6/18.4 | 7.0/6.8 | 1.7/1.6 | 16.4/16.1 |
| **8** | $[ZnS]_n$ | white | 21.2/20.9 | 7.8/7.5 | 0.7/0.6 | 18.2/18.1 |
| **9** | $[CdS(OH_2)_2]_n$ | white | 28.8/28.5 | 6.1/5.9 | 1.5/1.2 | 14.3/14.2 |
| **10** | $[CoS(NH_3)_2]_n$ | black-violet | 17.6/17..3 | 7.1/6.9 | 2.4/2.3 | 25.0/24.8 |
| **11** | $[NiS(NH_3)_2]_n$ | deep blue | 17.5/17.1 | 7.1/6.8 | 2.4/2.0 | 25.1/24.8 |
| **12** | $[CuS(NH_3)_2]_n$ | blue | 18.7/18.5 | 7.0/6.7 | 2.3/2.0 | 24.7/24.6 |
| **13** | $[ZnS(NH_3)_2]_n$ | white | 19.1/18.8 | 7.0/6.8 | 2.3/2.2 | 24.6/24.2 |
| **14** | $[CdS(NH_3)_2]_n$ | white | 28.9/28.5 | 6.1/5.7 | 2.0/2.1 | 21.6/21.3 |

[a]By gravimetry; [b]By combustion

The prepared derivatives were further characterized by spectroscopic, thermogravimetric and conductimetric measurements. In Table II some of these data are shown, more specifically the sulfonamido vibrations in the IR spectra of the ligand **4** and complexes **5-14**, as well as electronic absorption bands, thermogravimetric (TG) analysis and conductimetric data of these compounds.

Table II: Spectroscopic, magnetic and conductimetric data for complexes **5-10** as compared to ligands **4**.

| **Comp.** | **IR. Spectra** [a] ,$cm^{-1}$ $\nu(SO_2)^s$ | $\nu(SO_2)^{as}$ | **Electronic Spectra** [b], $\nu$ ($cm^{-1}$) | **TG** [c] calc./found | **Conductimetry** [d], $\Lambda_M$ ($\Omega^{-1}$ x$cm^2$x$mol^{-1}$) |
|---|---|---|---|---|---|
| **4** | 1107 | 1378 | - | - | 34 |
| **5** | 1088 | 1351 | 8,300; 17,500; 20,400 | 11.1/10.7 [e] | 54 |
| **6** | 1087 | 1348 | 9,450; 15,800; 26,300 | 10.7/10.5 [e] | 28 |
| **7** | 1087 | 1350 | 16,200 | 10.8/10.5 [e] | 18 |
| **8** | 1088 | 1347 | - | - | 21 |
| **9** | 1085 | 1357 | - | 9.2/9.1 [e] | 27 |
| **10** | 1086 | 1361 | 8,850; 17,800; 20,300 | 10.2/10.1 [f] | 29 |
| **11** | 1085 | 1360 | 9,100; 16,000; 26,300 | 10.1/9.9 [f] | 36 |
| **12** | 1088 | 1359 | 16,200 | 10.0/9.6 [f] | 31 |
| **13** | 1086 | 1359 | - | 10.2/9.9 [f] | 22 |
| **14** | 1087 | 1358 | - | 8.7/8.5 [f] | 30 |

[a] FTIR spectra of thin films of pure compound; [b] Diffuse reflectance spectra in MgO as reference; [c] Weight loss between 110-180°C for compounds **5-9**, and 70-120°C for compounds **10-14**; [d] Solution $10^{-3}$ M, in DMF, at 25°C; [e] Corresponding to 2 $H_2O$ for a metal ion; [f] Corresponding to 2 $NH_3$ for a metal ion.

In the FTIR spectra of **4** the strong $SO_2$ vibrations were evidenced at 1107 (symmetrical) and 1378 (asymmetrical) $cm^{-1}$, respectively, whereas $NH_2$ stretching vibrations appeared at 3080 $cm^{-1}$. For complexes **5-14** little differences were evidenced in the IR spectra, as compared to those of the ligand **4**, and they consisted in: (i) shifting of the two sulfonamide bands, with about 20-30 $cm^{-1}$ towards lower wavenumbers as compared to the corresponding bands of the ligand. This behavior was well documented for other complexes of heterocyclic sulfonamides, such as **1-3**, previously reported, [1,4,6,13,14] and clearly indicates that the deprotonated sulfonamido moiety interacts with the metal ions, a fact confirmed by X-ray crystallographic studies of several Zn(II) and Cu(II) complexes containing acetazolamide **1**, methazolamide **2** or other sulfonamides of this type [4c,11,13,14] ; (ii) the shift of the $\nu(NH_2)$ vibration at 3020-3030 $cm^{-1}$, in the spectra of the sodium salt of **4** as well as in that of complexes **5-14** (data not shown); (iii) in the spectra of the ammonia-containing complexes, **10-14**, such an additional band appears at 3345-3350 $cm^{-1}$, due to coordinated $NH_3$ molecules; (iv) the presence of bands around 340 and 399 $cm^{-1}$ in the spectra of complexes **5-14**, tentatively assigned as due to M-N and M-O vibrations. [13,14,15]

In the diffuse reflectance spectra of the Co(II) complexes **5** and **10**, an intense band with three maxima, around 8,500; 17,500, and 20,300 $cm^{-1}$, respectively was evidenced, which is characteristic for this ion in octahedral geometry.[16,17] The two Ni(II) complexes, **6** and **11**, present a similar spectrum, again with three maxima, around 9,100-9,500; 15,800-16,000 and 26,300 $cm^{-1}$, respectively, which is typical for Ni(II) in octahedral surrounding. [18] For the two Cu(II) complexes, **7** and **12**, a structureless wide band centered at 16,200 $cm^{-1}$ was observed, similarly with that of the acetazolamide complexes of Cu(II) previously reported by us [4a] and by Borras' group,[14] which contain Cu(II) in octahedral geometry. Probably derivatives **7** and **12** are octahedral too, as other Cu(II) complexes with this type of ligands, described by Raper. [19]

The $^1H$- and $^{13}C$-NMR spectra of ligand **4** are very simple due to the high symmetry of the molecule. A broad signal at 7.20 ppm due to the $SO_2NH_2$ protons is seen in the $^1H$-NMR spectrum, and one signal at 162.4 ppm in the $^{13}C$-NMR spectrum. [11] For the Zn(II) complexes **8** and **13** no signals were detected around 7 ppm in the proton spectra, whereas in the $^{13}C$-NMR spectra the signal was shifted at 169.5 ppm (for **8**), and 171.2 ppm (for **13**), respectively, possibly due to the neighborhood of the cation. Although few NMR data were published for complexes of sulfonamides, a similar behavior was documented by Casanova for the Zn(II) derivative of sulfathiazole. [20]

From the conductimetric data of Table II one can see that all the prepared complexes, as well as the original ligand, behave as non-electrolytes, in contrast to the disodium salt which is a 2:1 electrolyte (data not shown).

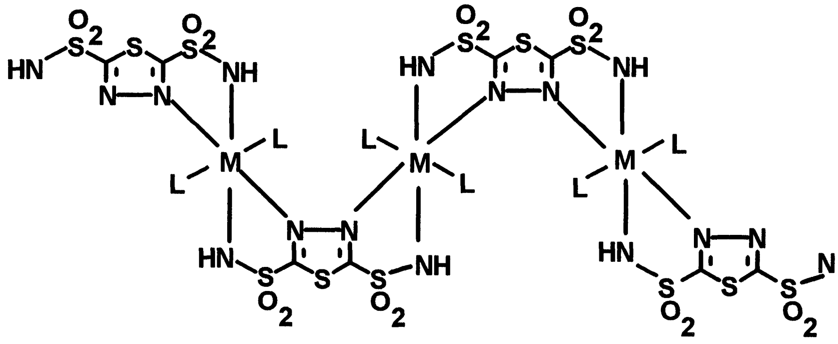

**5-7,9 : L = $OH_2$ ; 8 : no L present**

**10-14 : L = $NH_3$**

Correlating the above data with the TG analysis (Table II), which proved that two water and two ammonia molecules per metal ion, respectively, are also coordinated to these (with one exception, which is the Zn(II) derivatives **8**), one can presume that the geometry of central ions in the prepared complexes is tetrahedral for the Zn(II) derivative **8**, and octahedral (with two coordinated water or ammonia molecules) for all other derivatives. Considering **4** as a tetradentate ligand, by means of the two ionized sulfonamido nitrogens, and the two endocyclic nitrogens (as for the related bidentate ligand, acetazolamide **1**, previously investigated[5]) the polymeric structures shown above are proposed for the synthesized complexes, which would also explain their very low solubility in the majority of solvents. Practically. in contrast to other sulfonamide complexes,[5] derivatives **5-14** have a moderate or very low solubility in DMSO and DMF too.

Complexes **5-14** prepared in this study were tested for their ability to inhibit carbonic anhydrase ( (human isozyme I and bovine isozyme II were used in these assays). In Table III $IC_{50}$ values are presented for these compounds, comparatively with the corresponding data of the ligand **4** as well as the standard CA inhibitors, acetazolamide **1** and methazolamide **2**.

Table III: CA I and II inhibition data, for $CO_2$ hydration, with compounds **1-14**, determined by Maren's method. [10]

| Compound | $IC_{50}$ (nM)[a] CA I | CA II |
|---|---|---|
| **1** | 200 [b] | 7 [b] |
| **2** | 10 [c] | 8 [c] |
| **4** | $3.5.10^5$ | $10^4$ [d] |
| **5** | 345 | 30 |
| **6** | 390 | 45 |
| **7** | 175 | 24 |
| **8** | 210 | 28 |
| **9** | 180 | 17 |
| **10** | 360 | 42 |
| **11** | 440 | 41 |
| **12** | 185 | 27 |
| **13** | 205 | 22 |
| **14** | 185 | 19 |

[a] Molarity of inhibitor (calculated as for the monomer for complexes) producing a 50% decrease of enzyme specific activity for the $CO_2$ hydration reaction, at 0°C; [b] From refs. [1,3a]; [c] From ref. [21]; [d] From ref. [11].

Two things are very striking regarding data of Table III. First, the very weak inhibitory properties of disulfonamide **4** towards both CA isozymes investigated (data on CA I were not published before). Although structurally related to acetazolamide **1** and methazolamide **2**, the disulfonamide **4** is $10^3$-$10^4$ times a weaker CA inhibitor towards both these isozymes, as compared to the clinically used derivatives **1** and **2**. Thus, as shown earlier on CA II,[11] and confirmed here for the other red cell isozyme CA I, **4** has inhibitory properties comparable to those of benzene-1,3-disulfonamide. The other surprising fact is that, in contrast to **4** , its metal complexes prepared by us, of the type **5-14**, are much stronger CA inhibitors, a characteristic shared with all other complexes of heterocyclic sulfonamides reported in the last period by this group. [1,5] As shown in the introduction, this is due to the dual mechanism of inhibition of such compounds. Activity is dependent on the metal ion present in these compounds, with Cu(II), Cd(II) and Zn(II) derivatives being the most effective, towards both CA isozymes investigated.

In conclusion, this is the first report of metal complexes of a heterocyclic disulfonamide, which possess very strong CA I and CA II inhibitory properties, although the ligand itself, a historically important molecule for the development of diuretic agents, is weakly inhibitory. The new compounds prepared were characterized by standard procedures, and a polymeric structure was proposed for all of them. Unfortunately, due to their poor solubility in usual solvents, no good crystal for X-ray diffraction experiments could be obtained.

### References

1. C.T.Supuran, in "Carbonic Anhydrase and Modulation of Physiologic and Pathologic Processes in the Organism", I.Puscas ed., Helicon, Timisoara 1994, pp. 29-111.
2. T.H.Maren, *J.Glaucoma*, **1995**, *4*,49-62.
3. a)T.H.Maren, *Physiol.Rev.*, **1967**, *47*, 595-781: b) T.H.Maren, *Drug Dev.Res.*, **1987**, *10*, 255-276.
4. a)C.T.Supuran, , *Roum.Chem.Quart.Rev.*, **1993**, *1*, 77-116; b) T.H.Maren, B.W.Clare and C.T.Supuran, *Roum.Chem.Quart.Rev.*, **1994**, *2*, 259-282.
5. G.Alzuet, S.Ferrer, J.Borras and C.T.Supuran, *Roum.Chem.Quart.Rev.*, **1994**, *2*, 283-300.

6. a) C.T.Supuran, M.Andruh, and I.Puscas, *Rev.Roum.Chim.*, **1990**, *35*, 393-398; b) C.T.Supuran, G.Manole and M.Andruh, *J.Inorg.Biochem.*, **1993**, *49*, 97-104: c) L. Sumalan, J.Casanova, G.Alzuet, J.Borras, A.Castineiras and C.T.Supuran, *J.Inorg.Biochem.*, *in press:* d) C.T.Supuran, *Metal Based Drugs*, *in press.*
7. C.Luca, M.Barboiu and C.T.Supuran, *Rev.Roum.Chim.*, **1991**, *36*, 1169-1173.
8. D.N.Silverman and S.Lindskog, *Acc.Chem.Res.*, **1988**, *21*, 30-36.
9. R.O.Roblin and J.W.Clapp, *J.Am.Chem.Soc.*, **1950**, *72*, 4890-4892.
10. K.H.Beyer and J.E.Baer, *Pharmacol.Rev.*, **1961**, *13*, 517-562.
11. C.T.Supuran, C.W.Conroy and T.H.Maren, *Eur.J.Med.Chem.*, in press.
12. T.H.Maren, *J.Pharmacol.Exp.Ther.*, **1960**, *130*, 26-29.
13. a) C.T.Supuran, *Rev.Roum.Chim.*, **1993**, *38*, 229-236; b) G.Manole, O.Maior and C.T.Supuran, *Rev.Roum.Chim.*, **1993**, *38*, 474-479; c) C.T.Supuran and G.L.Almajan, *Main Group Met.Chem.*, **1995**, *18*, 347-351.
14. G. Alzuet, J.Casanova, J.Borras, J.A.Ramirez and O.Carugo, *J.Inorg.Biochem.*, **1995**, *57*, 219-234.
15. K.Nakamoto, "Infrared and Raman Spectra of Inorganic and Coordination Compounds", 3rd Edition, Wiley, New York, 1978, pp. 221-228.
16. I.Bertini, G.Canti, C.Luchinat and A.Scozzafava, *J.Am.Chem.Soc.*, **1978**, *100*, 4873-4877.
17. I.Bertini and C.Luchinat, in "Advances in Inorganic Biochemistry", G.L.Eichhorn and L.G.Marzilli Eds., Elsevier, New York, 1985, pp.148-159.
18. A.B.P.Lever, "Inorganic Electronic Spectroscopy", Second Edition, Elsevier, Amsterdam, 1984.
19. E.S.Raper, *Coord.Chem.Rev.*, **1994**, *129*, 91-156.
20. J. Casanova, Ph.D.Thesis, Univ. Valencia (Spain), 1995, pp. 170-172.
21. T.H.Maren, G.C.Wynns and P.J.Wistrand, *Mol.Pharmacol.*, **1993**, *44*, 901-905.

**Received: October 10, 1995 - Accepted: November 8, 1995 - Received in revised camera-format: November 21, 1995**

